# Reply to: The definition of locally advanced pancreatic cancer

**DOI:** 10.1038/sj.bjc.6605631

**Published:** 2010-03-30

**Authors:** S Boeck, V Heinemann

**Affiliations:** 1 Department of Internal Medicine III, Klinikum Grosshadern, Ludwig-Maximilians-University of Munich, Munich, Germany

**Sir,**

We would like to thank Zanini *et al* (2010) for their critical discussion of our data from a randomised phase II trial in locally advanced pancreatic cancer (LAPC) that was recently published in this journal (Wilkowski *et al*, 2009).

During the previous years, LAPC has been increasingly recognised as a separate disease entity in malignant pancreatic disorders. With the use of systemic gemcitabine-based chemotherapy, LAPC has a favourable survival prognosis compared with metastatic pancreatic cancer; however, the value of chemoradiotherapy still remains controversial in this stage of disease (Chauffert *et al*, 2008). We fully agree with Zanini *et al* that a unique, internationally standardised and accepted definition of LAPC is not yet established to date, and that such an approach is strongly recommend for future clinical trials in LAPC. Currently, different study groups still apply individual definitions for LAPC (Chauffert *et al*, 2008; Crane *et al*, 2009) and this definition probably is still mostly influenced by the definition of ‘resectability’ of the involved surgeon. A first step for the formulation of standardised criteria defining the respectability status in pancreatic cancer (e.g., ‘resectable’ *vs* ‘borderline resectable’ *vs* ‘unresectable’) is included in the current version of the NCCN guidelines for pancreatic adenocarcinoma (http://www.nccn.org, V.I.2009).

At the time when our phase II study was designed in 2001, the criteria for defining LAPC included in the study protocol were a first attempt to standardise the LAPC definition in the setting of a prospective multi-centre clinical trial. However, the authors are fully aware of the limitations of such an approach, as critically formulated by Zanini *et al.* In fact, much more future research will be necessary in order to better characterise LAPC clinically and biologically. Besides, currently – as recently reported in a study by Morak *et al* – the classification of a tumour as LAPC may still depend on the diagnostic possibilities and on the experience of a clinical institution (Morak *et al*, 2009).
